# Phylogenetic analysis and expression profiling of the Klotho gene family in the short-lived African killifish *Nothobranchius furzeri*

**DOI:** 10.1007/s00427-018-0619-6

**Published:** 2018-09-03

**Authors:** Gordin Zupkovitz, Julijan Kabiljo, David Martin, Sylvia Laffer, Christian Schöfer, Oliver Pusch

**Affiliations:** Center for Anatomy & Cell Biology, Medical University of Vienna, Schwarzspanierstr. 17, 1090 Vienna, Austria

**Keywords:** Klotho, African killifish, *Nothobranchius furzeri*, Aging

## Abstract

Members of the Klotho gene family have been identified as modulators of the aging process. Deletion of *αklotho* in the mouse results in a syndrome resembling rapid human aging. Conversely, overexpression of *αklotho* extends mammalian lifespan. Here, we identify *klotho* orthologs in the vertebrate aging model *Nothobranchius furzeri* and provide a detailed spatio-temporal expression profile of both paralogs, *α* and *βklotho*, from embryogenesis until old age spanning the entire life cycle of the organism. Specifically, we observe low levels of expression of both paralogs during embryogenesis followed by a significant transcriptional induction as development proceeds. In adult killifish, *αklotho* is predominantly expressed in the liver, the kidney, and the developing pharyngeal teeth. Particularly high levels of *α*Klotho protein were identified in the kidney tubules, closely resembling mammalian expression patterns. Prominent *βklotho* expression was detected in the killifish intestine and liver. Overall, qRT-PCR analysis of Klotho members as a function of age revealed steady transcript levels, except for *βklotho* expression in the liver which was significantly downregulated with age. This spatio-temporal expression profiling may serve as a useful starting point to further investigate the distinct physiological roles of Klotho members during the aging process.

**Electronic supplementary material:**

The online version of this article (10.1007/s00427-018-0619-6) contains supplementary material, which is available to authorized users.

## Introduction

Members of the Klotho gene family, including *α* and *βklotho*, have been described to play a critical role in the regulation of the aging process. The *αklotho* gene, named after the Greek goddess Klotho who spins the thread of life, was identified in 1997 as a gene mutated in a mouse strain that exhibited short lifespan and complex phenotypes resembling human premature aging syndrome (Kuro-o et al. 1997). Conversely, overexpression of the *αklotho* gene in mice suppresses aging and extends lifespan (Kurosu et al. 2005). *α*Klotho is a type I transmembrane protein that is primarily expressed in the brain and kidney (Kuro-o et al. 1997). The *α*Klotho protein exists in both membrane-bound and secreted forms exerting distinct functions. Membrane Klotho interacts with fibroblast growth factor (FGF) receptors to form high-affinity receptors for FGF23 (Urakawa et al. 2006) regulating phosphate metabolism. In addition to functioning as a co-receptor, the secreted form serves as a humoral factor. The secreted protein can enter the circulation via two pathways: either through an alternative splicing event or through shedding of the extracellular domain of the transmembrane protein by membrane-anchored proteases releasing the secreted form into blood, urine, and cerebrospinal fluid (Imura et al. 2004). Database mining identified *βklotho* as a gene homologous to *αklotho* (Ito et al. 2000). Similar to *αklotho*, *βklotho* encodes a single-pass transmembrane protein that forms complexes with FGF receptors (Ogawa et al. 2007). Despite the high structural similarity between *αklotho* and *βklotho*, their expression profiles vary considerably, with *βklotho* being predominantly expressed in the liver, pancreas, and adipose tissue of adult mice (Ito et al. 2000).

In the last decade, the turquoise killifish *Nothobranchius furzeri* has emerged as an exciting new model system for experimental aging research because it uniquely combines an exceptional short lifespan with vertebrate-specific features that are missing from currently used non-vertebrate organisms (Harel et al. 2015). With a lifespan of 4–6 months, *N. furzeri* is to date the vertebrate species with the shortest lifespan that can be bred in the laboratory (Valdesalici and Cellerino 2003). Despite their short lifespan, the fish show typical aging-related phenotypes such as physiological and cognitive decay, expression of aging-related biomarkers, decline in fertility, and cancerous lesions (Di Cicco et al. 2011). Additionally, *N. furzeri* is also sensitive to environmental changes that affect aging in other species, such as temperature (Valenzano et al. 2006a), caloric restriction (Terzibasi et al. 2009), and a resveratrol-enriched diet (Valenzano et al. 2006b). Only very recently, the successful completion of a reference genome for the short-lived turquoise killifish has been reported (Reichwald et al. 2015; Valenzano et al. 2015). In the present study, we identified orthologs of the Klotho gene family in *Nothobranchius furzeri* and provide a detailed spatio-temporal expression analysis of *αklotho* and *βklotho* covering the entire lifespan of this organism.

## Materials and methods

### Ethics statement

All killifish experiments and procedures were performed according to the “Principles of laboratory animal care” as well as to ethical standards of the Ethics Committee of the Medical University of Vienna.

### Killifish care and maintenance

All experiments were performed on *N. furzeri* of the GRZ strain (kindly provided by Dario Valenzano, MPI Cologne). The strain was originally collected in Zimbabwe in 1968, has an extremely short lifespan of about 16 weeks, and is highly inbred. For mating and breeding adult, animals were group and housed in a recirculation system with integrated multi-phase filtration. Fish were fed twice daily with live red blood worms (*Chironomus*) and maintained at 28 °C on a 12-h light/dark cycle. Eggs were collected twice a week. Fertilized eggs were incubated in 1× Yamamoto’s embryo solution with daily monitoring. Once eyes were recognizable, embryos were transferred to peat moss until ready to hatch. Fry was fed on freshly prepared brine shrimp (*Artemia nauplii*) until 3–4 weeks of age. Adult animals subject to further analyses were kept in a stand-alone overflow custom-made system with a fish density of one fish per 2.8-l tank. Water changes were performed every other day. For tissue collection and preparation, fish were euthanized with MS-222 and cooled on crushed ice for 5 min before dissection. Whole fish or dissected organs were further processed for IHC or snap-frozen in liquid nitrogen and stored at − 80 °C for RNA isolation.

### Survival curve

To analyze survival rates, two independent hatches consisting of 52 and 61 (total 113) fish were combined. Mixed-sex groups were set up at 5 weeks of age when animals showed first signs of sex dimorphism. Initial fish densities in these trials were eight animals per 25-l tank. Tanks were inspected twice a day and dead animals were instantly recorded and removed. Based on these data, survival rates were computed on a weekly basis. During the trial, we did not adjust population density caused by declining group size. Survival is presented as percentage of alive fish at a certain age per total number of fish.

### Cloning of killifish *αklotho* and *βklotho*

**Fig. 1:**
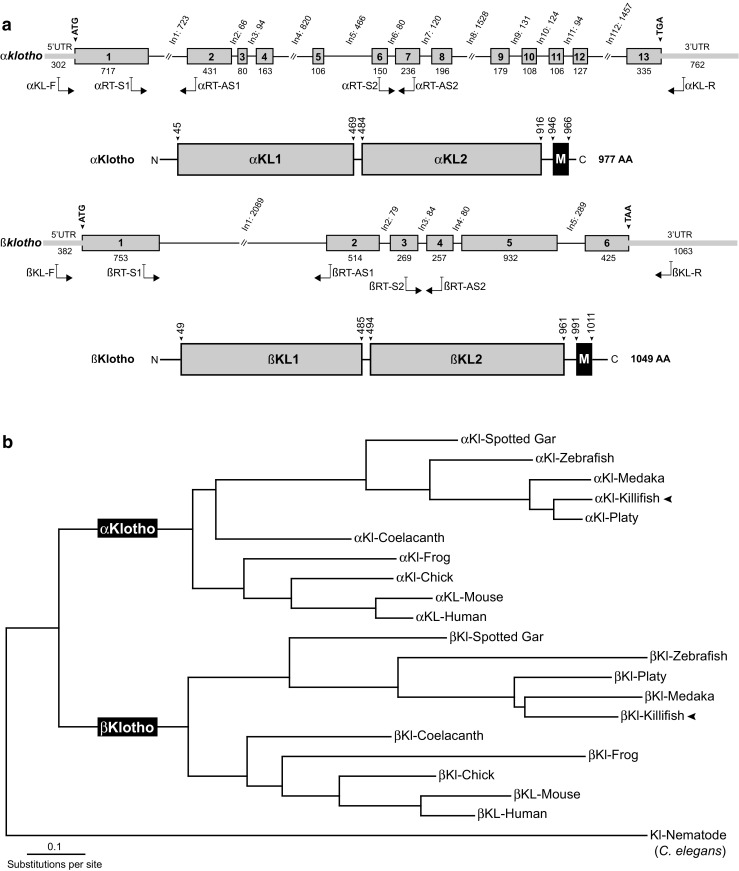
**a** Genomic organization of killifish orthologues of *klotho*. Boxes and lines represent exons and introns with the length of bp indicated. Translation initiation and termination codons are shown above. Gray lines specify 5′UTR and 3′UTR. Arrows denote the approximate binding sites of primers used for cloning and qRT-PCR. The predicted structural organization of the killifish Klotho proteins is illustrated below their respective genomic composition. Numbers above arrows indicate amino acid position. The proteins share a common structure with human and mouse homologs, consisting of an extracellular domain with two internal repeats (KL1 and KL2), a transmembrane domain (M), and a short intracellular part. **b** Phylogenetic tree based on protein sequences from the Klotho family. The tree was generated by the Maximum-Likelihood algorithm. Klotho protein from the nematode *C. elegans* was used as an outgroup. We considered the following species: human (*Homo sapiens*), mouse (*Mus musculus*), frog (*Xenopus tropicalis*), chicken (*Gallus gallus*) zebrafish (*Danio rerio*), spotted gar (*Lepisosteus oculatus*), medaka (*Oryzias latipes*), platyfish (*Xiphophorus maculatus*), killifish (*Nothobranchius furzeri*), and coelacanth (*Latimeria chalumnae*)

Killifish cDNA was synthesized from total RNA from 5-week-old fish using a combination of Oligo-dT and random primer with the GoScript Reverse Transcription System (Promega). To clone full-length killifish, *α* and *βklotho* PCR was performed with gene-specific primer pairs *α*KL-F, *α*KL-R and *β*Kl-F, *β*KL-R (Fig. 1a, listed in supplementary materials) using Q5 High-Fidelity DNA Polymerase (NEB). PCR products were cloned into the pJET1.2 blunt cloning vector (ThermoFisher) and constructs representing *α* and *βklotho* were subject to Sanger sequencing (Microsynth). Sequence analysis was performed using Geneious software.

### Sequence comparison and phylogenetic analysis

Protein sequences for alpha and beta Klotho family members were retrieved from the Ensembl genome database (Release 91; http://www.ensembl.org). We included the following species: human (*Homo sapiens*), mouse (*Mus musculus*), chicken (*Gallus gallus*), frog (*Xenopus tropicalis*), zebrafish (*Danio rerio*), spotted gar (*Lepisosteus oculatus*), medaka (*Oryzias latipes*), platyfish (*Xiphophorus maculatus*), and coelacanth (*Latimeria chalumnae*).

Killifish (*Nothobranchius furzeri*) sequences were obtained from the NFINgb genome browser (http://nfingb.leibniz-fli.de) (Reichwald et al. 2015). To artificially root the phylogenetic tree, we incorporated a Klotho homolog (C50F7.10) from the nematode *Caenorhabditis elegans*, which served as an outgroup. First, in order to obtain a high-quality multiple alignment, the sequences were compared using the muscle program (Edgar 2004). The phylogenetic tree was generated using the Maximum-Likelihood algorithm from the PhyML program (Guindon et al. 2009), applying the LG substitution matrix. The accession numbers of the sequences from which the protein sequences were retrieved are listed in supplementary Table 1.

### RNA isolation and quantitative real-time RT-PCR analysis

Total RNA was isolated from whole fish and the brain, liver, and muscle samples with TRI reagent (Sigma-Aldrich) as recommended by the manufacturer. One microgram of total RNA was reverse transcribed with the iScript cDNA synthesis kit (Bio-Rad) and real-time RT-PCRs were performed using KAPA SYBR Fast Universal Master Mix (Kapa Biosystems) on a Bio-Rad CXF384 qPCR machine. Primer sequences for cloning and qRT-PCR analysis can be found in supplementary Table 2.

### Immunohistochemistry

IHC analysis was performed as previously described (Murko et al. 2013). In brief, samples were fixed with 4% PFA, dehydrated, and embedded in paraffin by a routine embedding procedure. Four to five-micrometer sections were deparaffinized and rehydrated. Peroxidase activity was blocked using 1% H_2_O_2_ in 1xPBS for 15 min at room temperature. Samples, pretreated with heat-induced epitope retrieval (HIER) by steaming with citrate buffer for 30 min, were permeabilized with 0.5% Triton X-100 for 5 min at room temperature. After blocking, sections were incubated with a rabbit polyclonal anti-KL (Klotho) antibody (Sigma-Aldrich SAB2104815, immunogen: HRGYSIRRGLFYVDFLSQDKMLLPKSSALFYQKLIEKNGFPPLPENQPLE corresponding to amino acids 433–482 in the killifish *α*Klotho protein) diluted 1:200 overnight at 4 °C and then with a HRP-secondary antibody (Abcam) diluted 1:750 for 30 min at room temperature. Fluorescence signals were detected using the TSA Plus Fluorescence Kit (PerkinElmer) and counterstained with DAPI.

### In situ hybridization

Transcription templates for in situ hybridization probes were generated by PCR amplification with primers including T7 and T3 transcription initiation sites to synthesize antisense and sense (control) probes, respectively. Digoxigenin-labeled riboprobes were in vitro transcribed using the T7-T3 MaxiScript System (Ambion). Primer sequences can be found in the “Electronic supplementary material” section. In situ hybridization was performed as previously described (Murko et al. 2010). Briefly, samples were fixed with 4% PFA, dehydrated, and embedded in paraffin by a routine embedding procedure. Four to five-micrometer sections were deparaffinized and rehydrated. Before hybridization, slides were post-fixed with 4% PFA, treated with 0.2-M HCl for 10 min, followed by proteinase K digestion and dehydration. Air-dried sections were incubated with the probes, which were separately denatured at 92 °C for 5 min, in the hybridization mix overnight in a humidified chamber at 55 °C. The hybridization mix contained 2xSSC, 50% formamide, 10% dextran sulfate, 0.02% SDS, with or without 100 ng/μl salmon sperm DNA, and 100 ng/μl yeast t-RNA. After stringency washes in 2xSSC at 42 °C or at 60 °C for 10 min three times and in 2xSSC at room temperature for 10 min, blocking was done with 2% FCS for 30 min. Probes were detected with an alkaline phosphatase conjugated anti-digoxigenin antibody (Roche) diluted 1:500 and signals were developed using NBT/BCIP (Roche).

### Statistical analysis

Real-time PCR experiments were analyzed using CFX Maestro software and further graphically processed with Prism Graphpad software. The significance between groups was determined by the unpaired Student’s *t* test. *p* values were calculated with the Prism software and standard deviation (s.d.) is shown. *p < 0.05; **p < 0.01; ***p < 0.001.

## Results and discussion

### Evolutionary relationships of the Klotho family

To identify Klotho family genes in killifish, we screened the recently annotated genome of *N. furzeri* (Reichwald et al. 2015; Valenzano et al. 2015). Data mining of the publicly available databases (http://nfingb.leibniz-fli.de) produced two genes with significant homology to human and mouse orthologs of *αklotho* (Nfu_g_1_022108) and *βklotho* (Nfu_g_1_004524).

Killifish *αklotho* and *βklotho* are predicted to consist of 13 and 6 exons accounting for an open reading frame of 2934 bp (977 amino acids) and 3150 bp (1049 amino acids), respectively. To clone full-length killifish *klotho* orthologs, reverse transcription-polymerase (RT-PCR) was performed on cDNA from 5-week-old whole fish using gene-specific primer (Fig. 1a). Pairwise sequence comparison between killifish and human full-length Klotho proteins demonstrates a high degree of conservation, sharing 64% and 61% sequence similarity for *α*Klotho and *β*Klotho, respectively. Moreover, sequence similarities of the individual Klotho proteins between species are higher than homologies within the same species (Table 1). The protein alignment also revealed that the killifish Klotho orthologs encode the same type 1 transmembrane protein as its human counterpart, with two internal repeats KL1 and KL2 in the extracellular domain (Fig. 1a). In mammals, N-terminal signal sequences have been described for both Klotho paralogs (Kuro-o et al. 1997; Ito et al. 2000) which is apparently lacking in zebrafish *α*Klotho (Sugano and Lardelli 2011) and also in the two Klotho paralogs in the nematode *Caenorhabditis elegans* (Château et al. 2010). Using the protein prediction resource InterProScan (Finn et al. 2017), we found a strong putative signal sequence for both killifish orthologs *α*Klotho (AA 1–18) and βKlotho (AA 1–25). Notably, although killifish and zebrafish *α*Klotho share more than 80% sequence similarity, the N-terminal part does not show a significant degree of conservation (Supplementary Fig. 1). To examine the evolutionary relationships of killifish Klotho proteins within the vertebrate genomes, we performed phylogenetic analysis on amino acid sequences of Klotho homologs of human, mouse, chicken, xenopus, coelacanth, spotted gar, zebrafish, medaka, platy, and killifish. Coelacanth was included because of its status as a living fossil, representing sarcopterygian fish which transitioned to a terrestrial life, giving rise to modern tetrapods (Amemiya et al. 2013). The spotted gar, which diverged before the teleost-specific genome duplication, represents the unduplicated sister group of teleosts (Braasch et al. 2016). A phylogenetic tree employing the Maximum-Likelihood algorithm demonstrates that the different Klotho paralogs grouped explicitly within their distinct clade, displaying highest conservation with other teleost species.

**Table 1:** Pairwise sequence comparison between killifish and human Klotho gene family members. The percentage similarity was scored using Geneious software

	*α*KL-Human	*α*Kl-Killifish	*β*KL-Human	*β*Kl-Killifish
*α*KL-Human		*64.14%*	57.84%	54.51%
*α*Kl-Killifish	*64.14%*		55.81%	53.72%
*β*KL-Human	57.84%	55.81%		*61.40%*
*β*Kl-Killifish	54.51%	53.72%	*61.40%*	

Italics is used to emphasize the percentage similarity of orthologs comparing killifish with human

### Strong induction of *αklotho* and *βklotho* expression post hatching

**Fig. 2:**
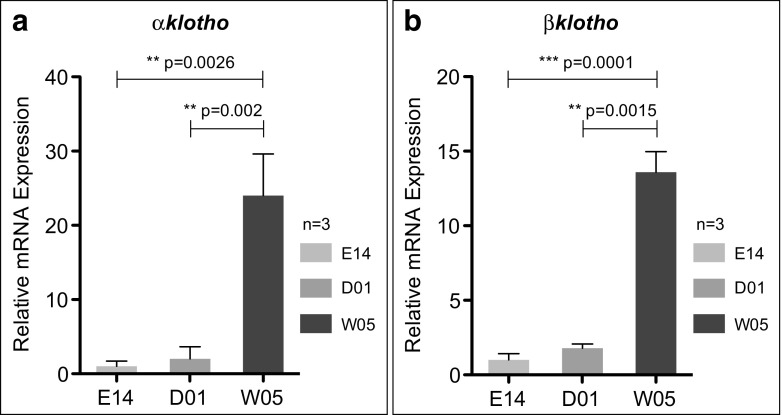
Expression of both **a** *αklotho* and **b** *βklotho* is strongly induced in adult fish. Relative expression was evaluated by qRT-PCR of *klotho* family members in *N. furzeri* whole fish cDNA extracts at embryonic day 14 (E14) corresponding to the “black eye” stage, post hatching day 1 (D01), and 5 weeks of age (W05) in triplicate. Gene expression was normalized to TATA-binding protein (TBP). All E14 data points are set to 1. Error bars represent ± SD. Student’s unpaired *t* test: ns *p* > 0.05; *p < 0.05; **p < 0.01; ***p < 0.001

To gain insight into the temporal expression profiles of Klotho family members, we compared three different time points during the early killifish life cycle by quantitative real-time PCR (qRT-PCR): embryonic day 14 (E14), shortly after hatching (D01), and week 5 (W05). Transcripts of both orthologs, *αklotho* and *βklotho*, were detected during embryogenesis. After hatching, a slight increase in mRNA expression was observed, followed by further strong induction in transcript levels within the first weeks of adult development (Fig. 2). These results agree with previous findings in zebrafish showing that *αklotho* transcripts were evident during early embryonic stages and gradually increased as development proceeded (Sugano and Lardelli 2011). Also in mouse, *α*Klotho immunoreactivity was initially observed at embryonic day 16 followed by an apparent induction after birth (Song et al. 2014). A similar temporal expression profile has been reported for murine *βklotho*, with transcription being progressively induced post-embryonic development (Ito et al. 2000). The fact that Klotho family members show only low levels of expression during embryogenesis in different vertebrate species suggests that *α*Klotho and *βKlotho* might not play an important role during embryonic development.

### Prominent expression of *αklotho* in the adult killifish liver and kidney

**Fig. 3:**
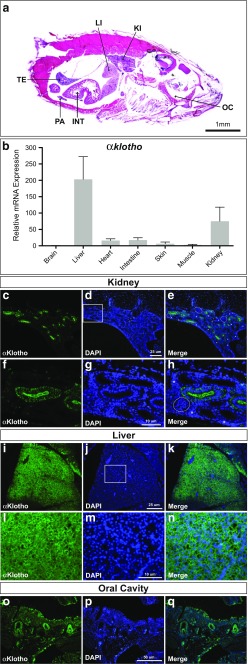
High expression levels of *αklotho* in the killifish liver and kidney. **a** Hematoxylin and Eosin staining of a sagittal section of 5-week-old fish. *INT* intestines, *LI* liver, *KI* kidney, *OC* oral cavity, *PA* pancreas, *TE* testis. **b** Relative expression of *αklotho* in multiple tissues was determined by qRT-PCR at time point 5 weeks of three biological replicates. Expression of *αklotho* was normalized to TATA-binding protein (TBP) gene expression. Fluorescence immunohistochemistry with an anti-*α*Klotho antibody showing *α*Klotho protein expression in the **c**–**h** kidney, **i**–**n** liver, and **o**–**q** oral cavity. Nuclei are counterstained with DAPI and merged with signals obtained from *α*Klotho-specific stainings. White boxes indicate regions shown in higher magnifications; dotted white lines encircle glomeruli; hematopoietic cells are marked with an asterisk

In mammals, the *αklotho* gene is expressed predominantly in the distal convoluted tubules of the kidney, the chief cells of the parathyroid gland, and the choroid plexus (Kuro-o et al. 1997) but has also been detected in additional tissues that were not included in the initial screens, such as reproductive organs (Li et al. 2004), the inner ear (Kamemori et al. 2002), the eye (Zhang et al. 2017), breast tissue (Wolf et al. 2008), bone cells (Kaludjerovic et al. 2017), and monocytes (Bacchetta et al. 2013). Also, a recent study combining immunohistochemistry with mass spectrometry reports widespread expression of transmembrane *α*Klotho in various human tissues (Lim et al. 2015). To characterize tissue expression of *αklotho* in killifish, qRT-PCR was performed on different tissues of 5-week-old fish (Fig. 5a). We detected *αklotho* expression in several adult tissues, being most prominent in the liver and kidney (Fig. 3b). To validate the significance of qRT-PCR data on the protein level, we performed immunostaining using an anti-*α*Klotho antibody on adult killifish tissues. Faint signal was detected in several tissues including the brain, testis, pancreas, and intestine while strong expression was observed in the kidney, liver, and pharyngeal teeth. A similar expression pattern has been reported in developing zebrafish (Mangos et al. 2012). Moreover, *αklotho* expression in *N. furzeri* is in line with the mammalian expression pattern. Particularly, high levels of protein were consistently identified in rat and mouse kidney tubules, whereas gene expression revealed weak signals in other organs including the brain (Kuro-o et al. 1997; Li et al. 2004). The morphology of the *N. furzeri* kidney has recently been described (Hoppe et al. 2015). Immunofluorescence distinctly highlighted tubules of nephrons, while no signal was found in glomeruli and hematopoietic cells (Fig. 3c–e). Subcellular signal predominately localized to cell membranes with the apical portion displaying the strongest signal (Fig. 3f–h). The lateral cell membranes were less strongly labeled but signal intensity increased towards the membranes of the baso-lateral compartment of tubular cells. Interestingly, the basal compartment appeared almost devoid of signal. Together, these findings corroborate results obtained from the mammalian kidney where *α*Klotho is expressed in specific sections of the tubules, namely predominantly in the distal convoluted tubules and not elsewhere in the kidney (Kuro-o et al. 1997; Song et al. 2014). Robust *α*Klotho protein expression was also observed in the liver of *N. furzeri* (Fig. 3i–k) with the signal predominately localized at hepatocyte cell membranes (Fig. 3l–n), concurring with its physiological function as a transmembrane protein. Prominent hepatic expression of *αklotho* was also reported in the liver of the longer-lived MZM 0410 strain of *N. furzeri* (GEO Accession number: GSE66712) whereas in mammals, it is *βKlotho*, which is primarily expressed in the liver tissue (Lim et al. 2015). Strong *α*Klotho expression was also detected in developing pharyngeal teeth of the oral cavity of *N. furzeri* (Fig. 3o–q). This expression site may be linked to mineralization of hard tissue considering the importance of *α*Klotho in electrolyte homeostasis. Indeed, mice lacking functional Klotho gene display tooth malformations (Hikone et al. 2017).

### Expression of *βklotho* in the adult killifish intestine and liver

Although *α*Klotho and *βKlotho* share high sequence homology and structural similarity, their reported tissue-specific expression patterns differ considerably, with *βklotho* being predominantly expressed in the liver, pancreas, and adipose tissue of adult mice (Ito et al. 2000).

**Fig. 4:**
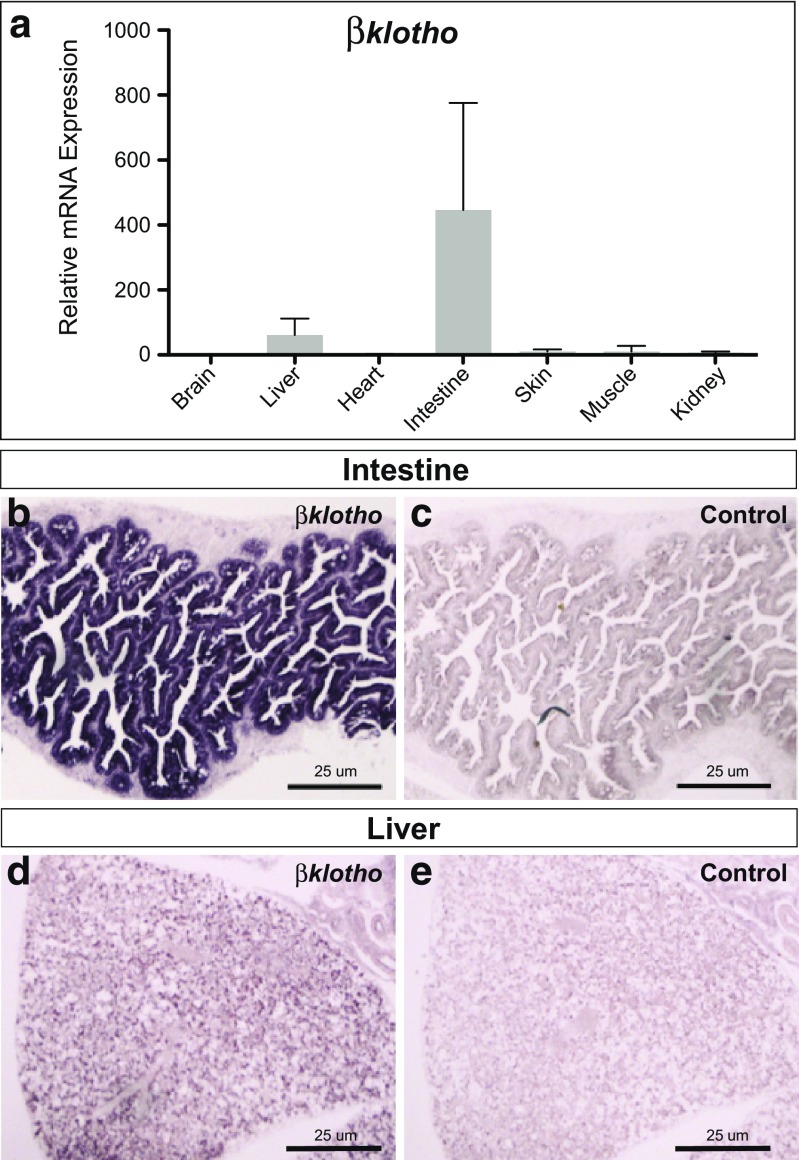
Expression of *βklotho* in the killifish intestine and liver**. a** Relative expression of *βklotho* in multiple tissues was determined by qRT-PCR at time point 5 weeks of three biological replicates. Expression of *βklotho* was normalized to TATA-binding protein (TBP) gene expression. Detection of *βklotho* mRNAs by in situ hybridization (ISH) in **b** intestine and **d** liver. **c**, **e** sense probes were used as controls

To investigate killifish *βklotho* expression in the spatial context, different tissues of 5-week-old fish were analyzed by qRT-PCR. *βklotho* transcripts were detected in the intestine and liver, with expression being particularly prominent in the intestinal tissue (Fig. 4a). In order to correlate qPCR data with morphology, we performed RNA in situ hybridization. Most prominent expression levels were identified in the intestine, where the signal was confined to the epithelial layer (Fig. 4b, c), and in the liver (Fig. 4d, e). Weaker expression was found in the kidney, pancreas, skeletal muscle, and testis (not shown). In general, the observed expression pattern closely resembles that in mice (Ito et al. 2000).

### Expression of *Klotho* family members during the aging process

**Fig. 5:**
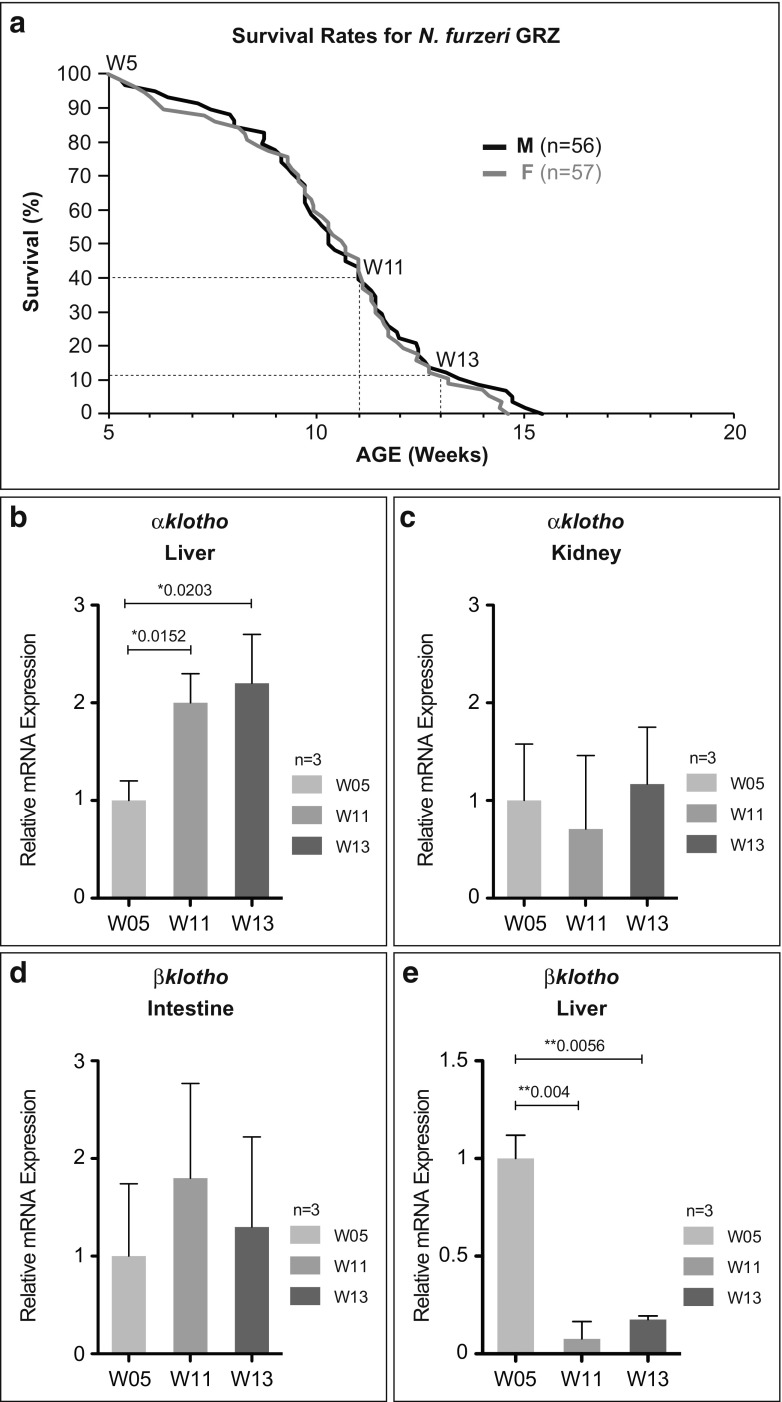
Relative gene expression profile of *klotho* family members during the aging process. **a** Age-dependent mortality of *N. furzeri* strain GRZ. Kaplan Meier estimates of survival for male (*n* = 56) and female (*n* = 57) populations. Total number of fish *n* = 113. Selected time points for RT-qPCR analysis are indicated. Temporal gene expression of *αklotho* in **b** liver and **c** kidney, and of *βklotho* in **d** intestines, and **e** liver comparing young (W05), aged (W11), and old (W13) *N. furzeri* GRZ was evaluated by RT-qPCR. Gene expression was normalized to TATA-binding protein (TBP). All W05 data points are set to 1. Error bars represent ± SD of three biological replicates. Student’s unpaired *t* test: ns *p* > 0.05; *p < 0.05; **p < 0.01; ***p < 0.001

Although numerous reports have addressed expression of Klotho members in different tissues and in the context of varying Klotho levels, there is limited information on temporal expression profiles during the aging process. A study in humans, comparing serum levels of secreted *α*Klotho between children and adults, demonstrated that the circulating form of *α*Klotho correlates negatively with age (Yamazaki et al. 2010). This decrease in *α*Klotho levels over time has also been observed in cerebrospinal fluid of humans. Additionally, the same study reports lower *α*Klotho levels in older adults with Alzheimer’s disease (Semba et al. 2014). Furthermore, it has been shown that both *αklotho* mRNA and protein expression are downregulated in brains of monkeys, rats, and mice with age. Of note, this age-dependent downregulation of *α*Klotho was only detected in white matter, as gray matter showed no significant differences in expression with age, suggesting cell type-specific regulation even within the same tissue (Duce et al. 2008). To characterize individual Klotho family members as a function of age, we performed qRT-PCR analyses of tissues showing prominent *αklotho* and *βklotho* expression (Figs. 3b and 4a). We compared three different time points, covering the entire killifish lifespan: week 5 (W05), week 11 (W11), and week 13 (W13). At W05, *N. furzeri* has reached sexual maturity, representing a young adult. Age W11 corresponds to approximately 40% survivorship in our colony (Fig. 5a). With survival rates having declined to 10–15%, W13 is defined as old age, indicated by a drastic decline in performance and display of typical aging-related phenotypes. Concerning *αklotho* regulation, we observed a gradual increase in transcription in the liver, whereas in the kidney, mRNA levels stayed unchanged during the aging process (Fig. 5b, c). These results are in accordance with a recent study characterizing age-related transcriptional changes comparing gene expression in kidneys of 14 and 96-week-old wild-type mice. Transcriptomic profiling revealed unchanged expression levels of *αklotho* during renal aging (Braun et al. 2016). In the case of *βklotho*, we found transcript levels to be steady in the intestine, whereas in the liver, a significant downregulation with age was observed (Fig. 5d, e). A recent study has shown that expression of *βklotho* was downregulated in human hepatocellular carcinoma tissues whereas its overexpression could inhibit tumorigenesis, hinting towards a role of *βKlotho* as a tumor suppressor in the liver (Ye et al. 2013). Histological analysis revealed high incidence of neoplastic lesions in the liver of *N. furzeri* with age (Di Cicco et al. 2011) suggesting a similar function of *βKlotho* in killifish. Decreased expression of *βklotho* in aged killifish liver might contribute to the observed phenotype.

Together, the high degree of conservation of the Klotho gene family in killifish and a tissue expression resembling mammals make *N. furzeri* an attractive experimental model system to further analyze pathways and molecular mechanisms that underlie the anti-aging properties of Klotho members. Work is ongoing to examine whether *α*Klotho overexpression reproduces lifespan expansion in this new model system.

## Electronic supplementary material

ESM 1 (PDF 522 kb)
