# Supplementary material for: Phylogenetic analysis and expression profiling of the Klotho gene family in the short-lived African killifish Nothobranchius furzeri
Source: Dev Genes Evol. 2018 Sep 3;228(6):255–65. doi: 10.1007/s00427-018-0619-6 (PMC6267267; doi:10.1007/s00427-018-0619-6)
Supplement: Supplementary file 1 — (PDF 522 kb) [file 427_2018_619_MOESM1_ESM.pdf]

Supplementary materials

Supplementary Figure 1

```
                                                                 KL1 ------>
αKL-Zabrafish   1 MKVTWIPLPLVLFCQFQGTASDPGAGQHTWDTFSKLPYPDDKAFLYDTFPDKFMWAVGTA 60
                  MK     + ++ F  F    + P AG  TW  FSKLPYP DKAFLYDTFP  F+WAVGTA
αKL-Killifish   1 MKGF---VAVLTFVAFAAVGAKPNAGLKTWSRFSKLPYPGDKAFLYDTFPGDFIWAVGTA 57
                      Signal Peptide
```

Supplementary Fig. 1. Sequence alignment of the N-terminal part of αKlotho protein between killifish and zebrafish using Geneious software. Putative signal peptide in killifish αKlotho identified by the protein prediction resource InterProScan is highlighted in grey. KL1 indicates the start of the first internal repeat in the extracellular domain. Sequence is highlighted in grey.

Supplementary Table 1

List of gene identifiers from which the protein sequences have been retrieved

**Human *(Homo sapiens)***
NP_004786.2
NP_783864.1
**Mouse *(Mus musculus)***
NP_038851.2
NP_112457.1
**Chicken *(Gallus gallus)***
XP_417105.2
XP_003641293.2
**Frog *(Xenopus tropicalis)***
XP_002934067.1
OCA48103.1
**Zebrafish *(Danio rerio)***
XP_021335093.1
XP_009305920.1
**Medaka *(Oryzias latipes)***
XP_011481593.1
XP_011478512.1
**Platy *(Xiphophorus maculatus)***
XP_014327768.1
XP_014325898.1
**Spotted Gar *(Lepisosteus oculatus)***
XP_006628277.1
XP_006630110.1
**Coelacanth *(Latimeria chalumnae)***
XP_006007832.2
XP_006011275.1
**Killifish *(Nothobranchius furzeri)***

Nfu_g_1_022108
Nfu_g_1_004524
**Nematode *(Caenorhabditis elegans)***
C50F7.10: Q18758

Supplementary Table 2
Primer Sequences

	Sequence (5'-3')
**Cloning, Sequencing, qRT-PCR**	
αKL-F	tccacggccagtaagaaaac
αKL-R	tgcagtacccacatttgacc
αRT_S1	atttttgcttccatgcgttt
αRT_AS1	gttgtagaggtgccatgcag
αRT_S2	cccagtttgcagaccctaac
αRT_AS2	tgagggaaaagtggaagtgg
ßKL-F	ctcctgcagagtggaaaagg
ßKL-R	tagaggggcgagtgtaggag
ßRT_S1	ttgttgtagcccacaatctga
ßRT_AS1	ctccatggactgctgacaaa
ßRT_S2	accaggacccctaaaaccac
ßRT_AS2	gggtcggtaaactgtggaga
**In situ hybridization**	
ßKL_ISH_S	gacaacggttttcctccaga
ßKL_ISH_AS	ggcctctagacccagcttct
T3-ßKL_ISH_AS	*AATTAACCCTCACTAAAGGG*gacaacggttttcctccaga
T7-ßKL-ISH_AS	*TAATACGACTCACTATAGGG*ggcctctagacccagcttct
